# Editorial for Special Issue “Genetic Basis and Epidemiology of Myopathies”

**DOI:** 10.3390/ijms22042152

**Published:** 2021-02-22

**Authors:** Eleni Peristeri, Efthimios Dardiotis

**Affiliations:** Department of Neurology, Faculty of Medicine, University of Thessaly, 41100 Larissa, Greece; edar@med.uth.gr

**Keywords:** myopathies, genetic basis, epidemiology

## Abstract

We are pleased to announce a Special Issue on the Genetic Basis and Epidemiology of Myopathies. This Special Issue is collecting papers pertaining to various lines of research focusing on the genetic basis and the epidemiology of myopathies. The Guest Editors’ note combines the contributing authors’ reviews and findings of relevant research, and we hope that future studies on myopathies will attempt to confirm these findings and, additionally, evaluate supplementary phenotypic and histological expressions of myopathies, as well as genetic factors in their pathogenesis.

## 1. Introduction

Congenital myopathies are a group of genetic muscle disorders clinically characterized by wide genetic and clinical heterogeneity. Identifying the candidate genes responsible for the various histological and phenotypic expressions of myopathies is of extreme importance for both highlighting the genetic basis of many forms of myopathies and improving treatment efficacy. While the genetic basis of myopathies has been extensively investigated, the genes involved in defects, especially cognitive ones, are still unknown.

## 2. Papers

This Special Issue of the *International Journal of Molecular Sciences* has attracted both research papers and reviews that contribute to our understanding of the genetic factors in the pathogenesis of myopathies and their epidemiology, their treatment, and their impact on the patients’ cognitive abilities. The first paper, titled “Description of a Novel Mechanism Possibly Explaining the Antiproliferative Properties of Glucocorticoids in Duchenne Muscular Dystrophy Fibroblasts Based on Glucocorticoid Receptor GR and NFAT5”, by [1], examines the antiproliferative properties of glucocorticoids in fibroblasts in Duchenne muscular dystrophy. Their results show the potential of glucocorticoids to slow down the process of fibrosis in Duchenne muscular dystrophy via modulating T-cells 5 (NFAT5) localization in the cell. localization in the cell. The second paper, titled “Abnormal NFAT5 Physiology in Duchenne Muscular Dystrophy Fibroblasts as a Putative Explanation for the Permanent Fibrosis Formation in Duchenne Muscular Dystrophy”, by [2] investigates the localization and expression of NFAT5 in skeletal fibroblasts in one patient with Duchenne muscular dystrophy and one healthy individual, after exposure to hyperosmolar or proinflammatory stress. Their findings show the unresponsiveness of NFAT5 to both hyperosmolar and proinflammatory stress in Duchenne muscular dystrophy, which may further explain the unchanged cell growth in fibroblasts. The third research paper, titled “Looking for Targets to Restore the Contractile Function in Congenital Myopathy Caused by Gln147Pro Tropomyosin”, by [3] explores the pathogenesis of skeletal muscle disease caused by the Gln147Pro substitution in β-tropomyosin through the technique of polarized microfluorimetry. According to their findings, a congenital myopathy-causing Q147P substitution in Tpm2.2 disrupts the myosin-induced displacement of tropomyosin over actin, which leads to the premature activation of actin monomers and increases the myosin cross-bridges in a state of strong binding with actin at low Ca^2+^. The fourth paper, titled “Preclinical Research in Glycogen Storage Diseases: A Comprehensive Review of Current Animal Models”, by [4] attempts a comprehensive review of animal models of glycogen storage diseases, including genetically modified mouse models, naturally occurring models and a genetically modified zebrafish model. The review highlights both differences and commonalities across the animal models, which contributes to better understanding the models’ advantages and deficiencies. The fifth paper, titled “Current Genetic Survey and Potential Gene-Targeting Therapeutics for Neuromuscular Diseases”, by [5] reviews studies that have focused on next-generation sequencing and genetic tools within the context of gene therapies for neuromuscular diseases. The review highlights both the interaction between mutations and the severity of the clinical phenotype, and the phenotype–genotype associations in neuromuscular diseases. The sixth paper, titled “Early-Onset Infantile Facioscapulohumeral Muscular Dystrophy: A Timely Review”, by [6] presents recent findings on the pathomechanisms underlying early-onset infantile facioscapulohumeral muscular dystrophy. The paper reviews patterns of muscle, respiratory and systemic involvement in infantile facioscapulohumeral muscular dystrophy and biomarkers of the disease, as well as therapeutic approaches and standard care for individuals with infantile facioscapulohumeral muscular dystrophy. The seventh paper, titled “Anti-Inflammatory and General Glucocorticoid Physiology in Skeletal Muscles Affected by Duchenne Muscular Dystrophy: Exploration of Steroid-Sparing Agents”, by [7] provides an overall presentation of the drugs that have been shown to stabilize the activation of proinflammatory and metabolic cellular pathways in skeletal muscle cells in Duchenne muscular dystrophy, as well as possible combinations of drugs with glucocorticoid therapy. The eighth paper, titled “Cognitive Deficits in Myopathies”, by [8] classifies myopathies according to their clinical characteristics and reviews concomitant cognitive deficits. The cognitive deficits in myopathies range from executive function difficulties to language and intelligence deficiencies across a wide range of age groups. The ninth paper, titled “Update on Congenital Myopathies in Adulthood”, by [9] classifies congenital myopathies following genetic-based approaches, with a special emphasis placed on late-onset congenital myopathies that first manifest in adulthood. Finally, the tenth paper, titled “New and Developing Therapies in Spinal Muscular Atrophy: From Genotype to Phenotype to Treatment and Where Do We Stand?”, by [10] presents an overview of the genotypes, phenotypes and recent therapeutic approaches for spinal muscular atrophy. The paper also highlights knowledge gaps in relevant research, as well as novel perspectives of recent therapeutics that are based on the identification of the pathomechanisms underlying spinal muscular atrophy.

## Data Availability

Not applicable.
